# Localization of subcentimeter pulmonary nodules using an indocyanine green near-infrared imaging system during uniportal video-assisted thoracoscopic surgery

**DOI:** 10.1186/s13019-021-01603-x

**Published:** 2021-08-06

**Authors:** Zhuo Wu, Lei Zhang, Xi-tong Zhao, Di Zhou, Xue-ying Yang

**Affiliations:** Department of Thoracic Surgery, The Fourth Affiliated Hospital of China Medical University, No.4, Chongshan East road, Shenyang, China

**Keywords:** Indocyanine green, Uniportal thoracoscope, Pulmonary nodules

## Abstract

**Background:**

To investigate the feasibility of indocyanine green (ICG) use in localizing subcentimeter pulmonary nodules during uniportal video-assisted thoracoscopic surgery.

**Methods:**

This study was a retrospective analysis of 32 patients who underwent surgery due to pulmonary nodules using ICG localization from September 2019 to March 2020 in the Department of Thoracic Surgery, The Fourth Affiliated Hospital of China Medical University. Laser positioning and large-aperture spiral CT simulation were performed preoperatively. ICG was injected into the lung (2.5 mg/ml). The clinical characteristics and postoperative indicators were recorded.

**Results:**

A total of 33 subcentimeter pulmonary nodules were successfully localized in 32 patients. Twenty-three patients underwent lobectomy, with an average surgical time of 45.3 min and an average tube retention time of 2 days. Non-small cell lung cancer was confirmed intraoperatively in 9 patients, among whom the longest surgical time was 120 min, and the shortest hospital stay was 7 days. No patient was converted to thoracotomy or developed serious complications.

**Conclusions:**

ICG imaging is a safe and effective technique for localization of pulmonary nodules. Due to the widespread application of near-infrared devices, fluorescent localization and imaging technology will be more widely used in thoracic surgery.

## Background

In recent years, due to the development of instruments and techniques for thoracic surgery, uniportal thoracoscopic surgery has been increasingly used in the diagnosis and treatment of lung and thoracic diseases. Globally, uniportal thoracoscopic surgery is considered to be a feasible and safe method for the treatment of pulmonary nodules and is commonly used [1, 2]. Consequently, localizing pulmonary nodules by tactile sense is no longer suitable due to the implementation of minimally invasive surgery. In addition, information provided solely by preoperative CT is insufficient to aid in the intraoperative detection of subcentimeter pulmonary nodules [3, 4]. Therefore, the intraoperative localization of small pulmonary nodules remains challenging for thoracic surgeons.

Indocyanine green (ICG) is a near-infrared fluorescent dye. When stimulated by an external light source, it emits near-infrared light with a wavelength of approximately 830 nm. ICG has been approved by the U.S. Food and Drug Administration (FDA) as a safe imaging agent and has almost no risk of allergic reactions (approximately 0.003%). As a highly sensitive and specific fluorescent imaging method, ICG imaging has been introduced for general use for the diagnosis and treatment of pelvic and abdominal tumors, tumor-node-metastasis, and ocular vascular and neurological diseases [5].

This study aimed to explore the safety and feasibility of an ICG fluorescent imaging system combined with laser positioning and CT simulation in intraoperative localization of subcentimeter pulmonary nodules.

## Methods

### Clinical data

A total of 32 patients admitted to the Department of Thoracic Surgery, The Fourth Affiliated Hospital of China Medical University from September 2019 to March 2020 were enrolled in this study. The inclusion criteria were (1) single or multiple pulmonary nodules with a diameter ≤ 1.5 cm; (2) nodules were located in the lung parenchyma and were ≤ 3 cm from the pleura, and preoperative CT images showed no pleural traction; and (3) no history of allergy to iodine contrast agents. All patients signed informed consent forms.

### Materials and devices

Injectable ICG (2.5 mg/ml) was purchased from Dandong Yichuang Pharmaceutical Co., Ltd., China. The near-infrared fluorescence endoscopic imaging system was purchased from Optomedic Co., Ltd., China. The large-aperture spiral CT and the laser positioning systems were purchased from General Electric Co., USA and Siemens AG, Germany, respectively.

### Surgical procedure

Forty minutes to one hour before the operation, a puncture was performed in the patient under laser positioning and CT guidance to a needle depth of 0.5–2 cm. The ICG agent (2.5 mg/ml, 0.3–1 ml) was injected slowly. The respiratory rate, blood pressure, and heart rate were monitored.

After general anesthesia was induced, a single 3–5 cm incision was made in the chest wall. After one-lung ventilation, the imaging system was switched to fluorescence detection to localize the nodule. A wedge excision was performed around the localized point. The sample was inspected by tactile sense and the naked eye after the surgery to verify the presence of pulmonary nodules. Based on the pathological results of the frozen section analysis, a decision was made regarding whether to perform pulmonary lobectomy.

## Results

### Patient Characteristics in ICG imaging

**Fig. 1:**
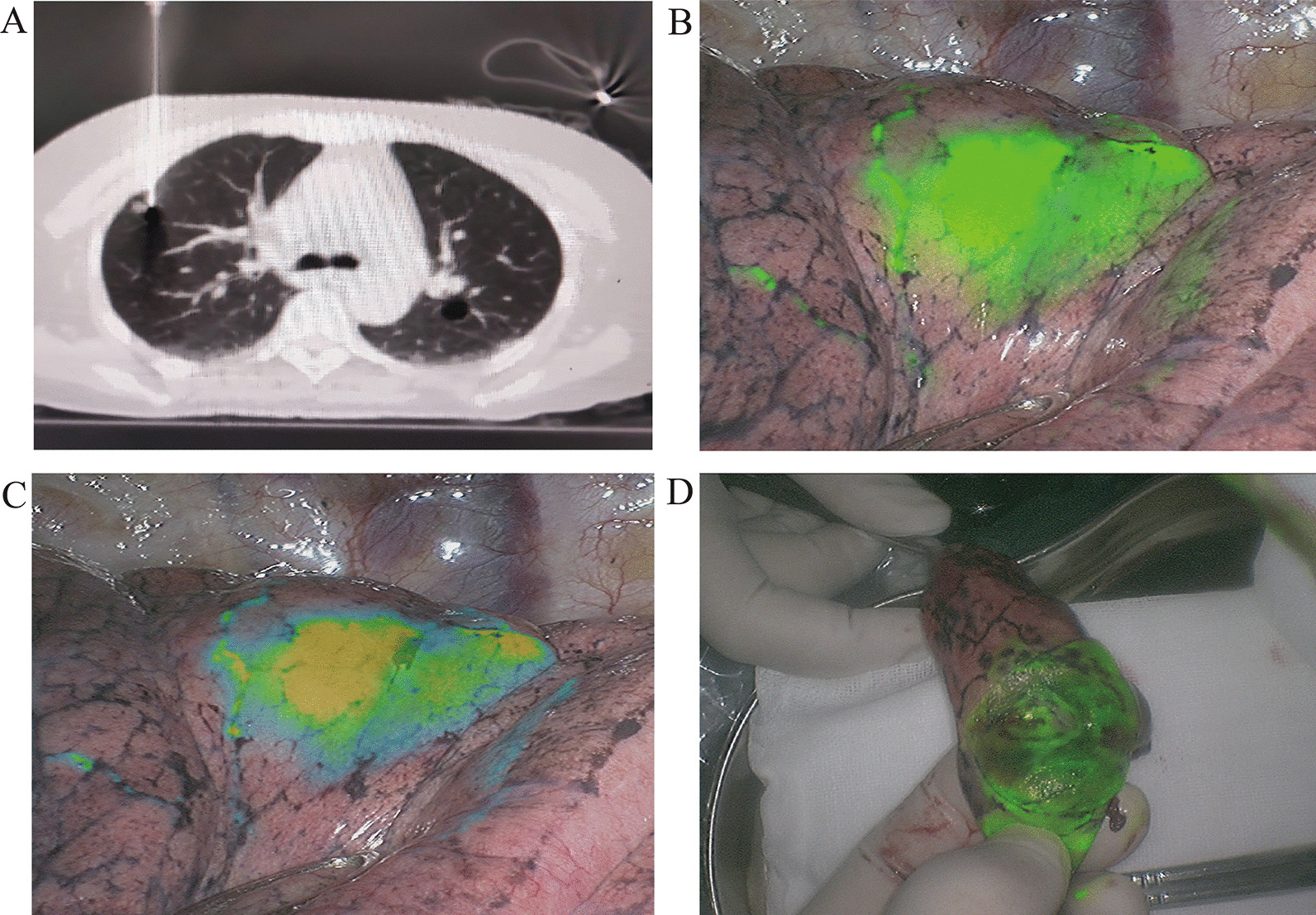
**a** Laser-guided, high-aperture, spiral CT localization. The localization needle was placed near the pulmonary nodule at a depth about 1 cm. **b** Localization and imaging under the standard fluorescence mode. Green fluorescence was visible on the surface of the lung. **c** Localization and imaging under the enhanced fluorescence mode. Yellow and blue fluorescence were visible. Yellow fluorescence was visible in the area where the indocyanine green concentration was highest. **d** Green fluorescence was visible in the sample under the standard fluorescence mode

**Fig. 2:**
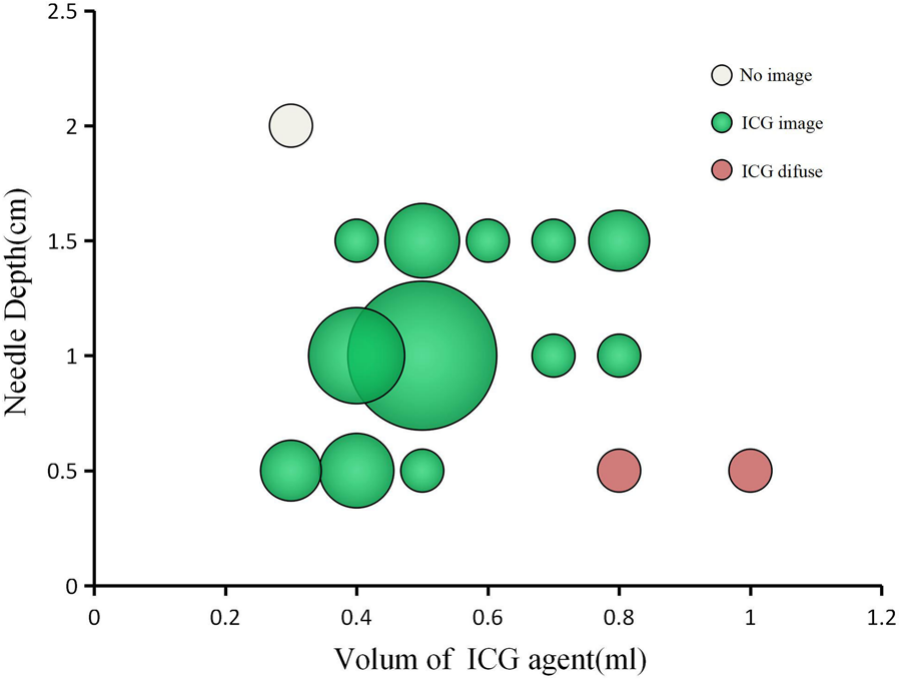
Bubbles of the different doses of indocyanine green at various puncture depths. The range of needle depth was 0.5–2 cm and ICG (2.5 mg/ml, 0.3–1 ml) was injected. The better images were obtained when 0.4–0.5 ml of ICG and 1 cm of needle depth

The mean age of the 32 patients was 59.2 years (range 43–79 years), including 15 males and 17 females. The mean forced expiratory volume in one second (FEV_1_) was 2.15L in the preoperative pulmonary function tests. A total of 36 nodules were excised, including 4 multiple pulmonary nodules at different sites that required multiple localization processes. The average nodule size was 0.76 cm (range 0.4–1.5 cm). The average time required for laser positioning and CT-guided localization was 8.3 min (6–15 min). Three patients with nodules at multiple sites who underwent multiple localization processes presented with different degrees of chest pain and dyspnea. The remaining 29 patients had no adverse reactions. Nodules were successfully localized in 32 cases. The nodules were not localized in 3 cases, including 2 cases with diffuse thoracic images and 1 case in which the images were not developed. In the standard fluorescence mode, green fluorescence was visible in the vicinity of the localized point. In the enhanced fluorescence mode, the central area with a high concentration of ICG displayed yellowish fluorescence, and the peripheral area displayed blue fluorescence (Figs. 1 and 2). Intraoperative pathology confirmed non-small cell lung cancer in 9 cases, for which lobectomy and lymph node sampling were performed. The remaining patients underwent wedge excision. The clinical characteristics and tumor parameters are shown in Table 1.

**Table 1:** Clinical characteristics and tumor parameters

Clinical characteristics	n (%)
Total number of patients	32
Male	15 (46.9%)
Female	17 (53.1%)
Age, years (range)	59.2 (43–79)
Nodule size, cm (range)	0.76 (0.4–1.5)
Pulmonary function: FEV_1_, L (range)	2.15 (1.23–3.56)
*Imaging characteristics of pulmonary nodules*	
Pure ground-glass nodules	9 (25%)
Partial solid nodules	12 (33.3%)
Solid nodules	15 (41.7%)
*Nodule location*	
Left upper lobe	6 (16.7%)
Left lower lobe	9 (25%)
Right upper lobe	7 (19.4%)
Right middle lobe	6 (16.7%)
Right lower lobe	8 (22.2%)
*Localization*	
Success	33 (91.7%)
Failure	3 (8.3%)
*Pathology*	
Non-small cell lung cancer	9 (25%)
Carcinoma in situ	5 (13.9%)
Atypical hyperplasia	12 (30%)
Organizing pneumonia	5 (13.9%)
Metastatic cancer	2 (5.6%)
Lymphoid tissue	3 (8.3%)

### Treatment effect after ICG imaging

The average surgical time of wedge excision of the lung was 45.3 min in 23 patients, and the surgical time in the case with localization failure was 62 min, which was significantly higher than the average time. No adverse reactions occurred postoperatively. Postoperative thoracic drainage was not necessary in 5 patients. The average tube retention time was 2 days. None of the 23 patients were converted to thoracotomy, and the shortest hospital stay was 4 days. Nine patients who were intraoperatively confirmed to have non-small cell lung cancer underwent uniportal thoracoscopic lobectomy and lymph node sampling. None of these patients were converted to thoracotomy, the longest surgical time was 120 min, and the shortest hospital stay was 7 days. One of the 9 patient developed atrial fibrillation due to difficulty in eliminating sputum. Atrial fibrillation disappeared after suctioning using a fiberoptic bronchoscope (Tables 2 and 3).

**Table 2:** Postoperative clinical characteristics of patients with wedge excision

Clinical characteristics	n (%)
Wedge excision	23 (71.9%)
Surgical time, minutes (range)	45.3 (38–62)
Intraoperative blood loss, ml (range)	30.4 (20–70)
Postoperative complications	0
Conversion to thoracotomy	0
Drainage tube retention time, days (range)	2 (0–5)
Hospital stay, days (range)	6.5 (4–10)

**Table 3:** Postoperative clinical characteristics of patients with lobectomy

Clinical characteristics	n (%)
Lobectomy + lymph node sampling	9 (28.1%)
Surgical time, minutes (range)	65 (50–120)
Intraoperative blood loss, ml (range)	110 (50–400)
Postoperative complications	1 (11.1%)
Conversion to thoracotomy	0
Drainage tube retention time, days (range)	3.5 (1–7)
Hospital stay, days (range)	9 (7–12)

## Discussion

Due to the improvement of health awareness and the increasing number of spiral chest CT examinations, the detection rate of intrapulmonary subcentimeter nodules is continuously increasing [6]. However, the wide use of thoracoscopic surgery leads to increasing difficulties in localizing pulmonary nodules by the conventional method of tactile sense. With the development of minimally invasive concepts, uniportal thoracoscopic surgery represents a new frontier for minimally invasive thoracic surgery [7, 8]. Given this basis, the new generation of thoracic surgeons are eager to further reduce surgical time and trauma, thereby providing better healthcare for patients. Thus, a more precise and effective localization method for uniportal endoscopic surgery is urgently needed [9]. Over time, different localization techniques, including the hookwire and microcoil methods, have been proposed, inevitably leading to complications such as dislodgement, systemic air embolism, pulmonary hemorrhage, and pain [10–12]. Colored dyes, including methylene blue, can easily contaminate operative areas and affect surgical exposure, and because of their small molecular weight, these dyes can easily diffuse, leading to incorrect intraoperative identification or excessive resection of nodules [13, 14].

The near-infrared dye ICG has been widely used in many fields. The Chinese Experts’ Consensus of Preoperative-assisted Localization of Pulmonary Nodules (2019 version) recommends localization methods using liquid materials, including ICG, as a 2A-level technique [15]. Rho J et al. [16] found that an optimal emulsion of 10% ICG and 90% lipiodol mixed through 90 passages exhibited an even distribution and the highest signal intensity under fluorescence microscopy and that all emulsion types injected were well localized around the target nodules without any side effects or procedure-related complications. Chao Zhan et al. [17] performed preoperative ICG localization for lung lobectomy in 11 patients, and the localization failed in 2 patients due to unclear fluorescent signals. They also found that for segmentectomy, ICG fluorescence was visible at 14 s after peripheral intravenous administration. They concluded that ICG fluorescence imaging could facilitate a precise segmentectomy and reduce the surgical time and trauma. Further studies demonstrated that near-infrared fluorescence imaging could safely identify lung tumors after systemic injection of ICG; in addition, low-dose ICG was adequate for near-infrared fluorescence imaging of lung tumors. However, as passive accumulation of ICG cannot be used to distinguish between tumors and inflammation, targeted fluorescent agents should be developed to address this problem [18].

This study showed that the optimal imaging effect was obtained when 0.4–0.5 ml of ICG (25 mg/ml) was administered at a needle depth of 1 cm. If the dose was too large or the needle depth was too shallow, ICG overflow could occur. An injection volume of less than 3 ml may not be sufficient for localization. If the injection is performed too quickly, ICG overflow may occur. In this study, 2 cases of ICG overflow were separately caused by an excessive dose and rapid injection. One case of localization failure was caused by an inadequate dose (no ICG reached the lung). The surgical time in the cases with localization failure was obviously prolonged. No patients had allergic reactions. Three patients reported pain after puncture. Complications such as pneumothorax and hemothorax did not occur.

We conclude that ICG imaging is a safe and effective technique for pulmonary nodule localization. Because of the short study time, small sample size, and lack of controls in this study, further observations and randomized controlled clinical trials should be conducted in the future. With the widespread application of near-infrared imaging devices, fluorescent imaging localization will become more widely used in thoracic surgery. Due to the continuous development of uniportal thoracoscopic surgical techniques, the combination of these two methods is expected to provide greater benefits for patients.

## Data Availability

The datasets used and analyzed during the current study are available from the corresponding author on reasonable request.

## Abbreviations

ICG: Indocyanine green
FDA: Food and Drug Administration
FEV_1_: Forced expiratory volume in one second
